#  The first report of *Enterobacter gergoviae* carrying *bla*_NDM-1_ in Iran

**DOI:** 10.22038/ijbms.2020.41225.9752

**Published:** 2020-09

**Authors:** Reza Khashei, Fatemeh Edalati Sarvestani, Yalda Malekzadegan, Mohammad Motamedifar

**Affiliations:** 1 Department of Bacteriology and Virology, School of Medicine, Shiraz University of Medical Sciences, Shiraz, Iran

**Keywords:** Antimicrobial resistance, β-lactamase, bla_NDM-1_, Carbapenems, Enterobacter

## Abstract

**Objective(s):**

Prompt detection of extended-spectrum β-lactamases (ESBL) and carbapenemase-producing enterobacteriaceae is crucial for infection prevention and control strategies. The present study aimed to characterize the ESBL and carbapenemase genes among *Enterobacter* isolates from an Iranian inpatient population.

**Materials and Methods:**

A total of 96 *Enterobacter* isolates obtained from inpatients between June 2016 and March 2017, were identified by the conventional microbiological methods and diagnostic kits. Antimicrobial susceptibility pattern was performed using the disk diffusion method. The ESBL and carbapenemase genes were screened using polymerase chain reaction (PCR).

**Results:**

All clinical isolates of *Enterobacter* were classified as *E. gergoviae* (52, 54.2%), *E. aerogenes* (34, 35.4%), *E. cloacae* (7, 7.3%), *Cronobacter* *(E). sakazakii* (3, 3.1%). The highest and lowest antimicrobial resistance rates were observed against ampicillin (93.8%) and imipenem (21.9%). High prevalence of multi-drug resistance (MDR=96.9%) was substantial. Of the 96 *Enterobacter* isolates, 35 (36.5%) and 28 (29.2%) were phenotypically ESBL-positive and non-susceptible carbapenem, respectively. Overall, the frequency of evaluated genes was as follows: bla_CTX-M_ =25 (26%), bla_TEM_ =30 (31.3%), bla_SHV_ =12 (12.5%), bla_IMP_ =3 (3.1%), bla_VIM_ =0 (0%), bla_NDM_ =8 (8.3%), and bla_KPC_ =0 (0%).

**Conclusion:**

In this study, we report for the first time the presence of *E. gergoviae* harboring bla_NDM_ from an Iranian population. Regarding the increase of MDR *Enterobacter* spp. in our region, strict hygiene rules will be needed to control the quick spread of ESBL and carbapenemase-producing *Enterobacter* isolates in healthcare facilities of developing countries.

## Introduction

Among enterobacteriaceae members, *Enterobacter* spp. is of particular concern, since it exhibits a higher level of resistance to antibiotics than other genera (1). The presence of β-lactamases, especially extended-spectrum β-lactamases (ESBLs) among Gram-negative bacteria is a major issue in clinical settings (2). The production of ESBLs is one of the most important mechanisms of resistance to extended-spectrum penicillins, third-generation of cephalosporins and monobactams, except for cephamycins and carbapenems (3-5). These enzymes have been reported in many enterobacteriaceae members, including *Enterobacter* spp (2, 3). The increasing prevalence of ESBL-producers is seen among both in and outpatients worldwide, ranging from 3–60% (2-4). The members of TEM, SHV, and CTX-M β-lactamases in *Klebsiella* and *Enterobacter* spp. are the most important ESBLs which have been growing all around the world (6).

Carbapenems are frequently used to treat infections due to cephalosporinase or ESBL-producing multidrug-resistant (MDR) Gram-negative rods such as *Enterobacter* species. However, the emergence of carbapenemases among these bacteria has restricted use of carbapenems in medical practice (7, 8). The main mechanism in emergence of carbapenem-resistant enterobacteriaceae (CRE), including *Enterobacter* spp., is the production of carbapenemases. Different carbapenemases have been described amongst these bacteria, including Ambler class A *bla*_KPC_*, *metallo-β-lactamases (MBL) class B such as *bla*_VIM_, *bla*_IMP_, *bla*_NDM_, etc. (9, 10).

Nosocomial infections caused by CRE are considered serious clinical challenges for physicians worldwide, and this issue is due to the capability of their rapid spread around the world. The mortality rate of infections caused by CRE is considerable, ranging from 30-44% (9-11). Moreover, infections caused by ESBL-producing bacteria, including *Enterobacter* spp., among inpatients are accompanied by increased mortality (12).

Reports about the prevalence of ESBL and carbapenemase-producing *Enterobacter* spp. from Iran are scarce. This study was undertaken to characterize infections caused by ESBL-positive and carbapenem-resistant *Enterobacter* spp. collected in Shiraz Namazi Hospital, Shiraz, Iran. 

## Materials and Methods

***Clinical isolates***

A total of 96 non-repetitive *Enterobacter* isolates were obtained from patients hospitalized at a university-affiliated medical center (Namazi) in Shiraz, Southwest of Iran from June 2016 to March 2017. Only one isolate was collected per patient. The isolates were recovered from different clinical samples, namely blood, wound, sputum, endotracheal tube aspirates, abdominal discharge, urine, and eye. *Enterobacter* spp. was initially identified by standard microbiological tests and confirmed using API 20E (bioMérieux, Marcy l’Etoile, France) and Microgene^TM ^GnA+B-ID system (Microgen Bioproducts Ltd, UK) diagnostic kits. Confirmed *Enterobacter *spp. isolates were stored in tryptic soy broth (TSB) (Merck Co., Germany) containing 20% glycerol at -70 ^°^C until further study. This study was in accordance with the declaration of Helsinki and ethical approval was sought from the institutional Ethics Committee of Shiraz University of Medical Sciences (approval No. EC IR.SUMS.REC.1396.S526). However, because we only used leftovers from clinical specimens, the local ethics committee waived the need for informed consent.

***Susceptibility testing***

Antimicrobial susceptibility pattern was determined by the disk diffusion method on Muller-Hinton agar plates (Merck Co., Germany) following Clinical and Laboratory Standards Institute (CLSI) guidelines (13). Guidelines of the CLSI were used for ampicillin, ceftazidime, cefoxitin, gentamicin, amikacin, ciprofloxacin, trimethoprim-sulfamethoxazole (SXT or Co-trimoxazole), nitrofurantoin, amoxicillin-clavulanate, and imipenem (Mast Co., UK). *E. coli* ATCC 25922 was used as the quality control strain. MDR was defined as non-susceptibility to ≥1 agent in ≥3 different antibiotic classes (14).

ESBL phenotypic detection was performed using the combination disk method in accordance with CLSI recommendations (13). All ceftazidime (as a third-generation cephalosporin) resistant isolates were selected for evaluation of ESBL production. In this test, ceftazidime (30 µg) and cefotaxime (30 µg) disks were applied alone and in combination with clavulanic acid (30/10 µg). An increase of ≥ 5 mm in the inhibition zone of the agent in combination with clavulanic acid was considered ESBL producer. *E. coli* ATCC 25922 and *Klebsiella pneumoniae* ATCC 700603 were used as negative and positive control strains, respectively. 

***Genotypic detection of ESBL and carbapenemase genes***

Genomic DNA was extracted from overnight TSB culture using a Cinna-pure kit (CinnaGen Co., Iran) according to the manufacturer^’^s instructions. Molecular characterization of ESBLs (*bla*_TEM_, *bla*_SHV_, and *bla*_CTX-M_) and carbapenemases (*bla*_KPC_*, bla*_VIM_, *bla*_IMP_, and *bla*_NDM_) were screened in all isolates by PCR amplification using specific previously reported primers (15, 16). PCRs were performed using a thermal cycler 5530 (Ependrof master, Germany) with 1 μl of each specific primer (1 μM), 3 μl DNA template, 2.5 μl PCR buffer (1X), 1 μl deoxyribonucleotide triphosphates solution (dNTPs, 200 μM), 1.5 μl MgCl_2_ (1.5 mM), and 0.25 μl Taq DNA polymerase (1 Unit) in a total volume of 25 μl. PCRs comprised 5 min at 94 ^°^C as initial denaturation, followed by 30 cycles of denaturation at 94 ^°^C for 30 sec, annealing (the eventual annealing temperatures chosen were 45–60 ^°^C for corresponding genes), extension of 1 min at 72 ^°^C, and a final elongation step of 7 min at 72 ^°^C. The positive PCR products were screened by electrophoresis on agarose 1.5% w/v gels and stained with safe stain load dye (CinnaGen Co., Iran) and visualized through UV transillumination.

***DNA sequence analysis***

To confirm the accuracy of amplified carbapenemase genes (one sample of each positive gene and three samples for *bla*_NDM _gene), the amplicons were submitted for sequencing (Bioneer Co., Munpyeongseoro, Daedeok-gu, Daejeon, South Korea) and the sequences were compared using online BLAST software (http://www.ncbi.nlm.nih.gov/BLAST/). For ESBL genes, *Klebsiella pneumoniae* ATCC 700603 was used as control strain.

***Statistical analysis***

The Chi-square (χ^2^) test was used to analyze significant differences between the studied resistance genes and the clinical outcome, using SPSS (ver. 21.0; IBM Co., Armonk, NY, USA) software. The results of demographic and clinical manifestations were presented as descriptive statistics in terms of relative frequency. A *P-*value<0.05 was considered as significant clinical relevance. 

## Results

***Study population and clinical characteristics of Enterobacter isolates***

The isolates were collected from 96 individuals admitted as inpatients, consisting of 62 (64.6%) men and 34 (35.4%) women with a median age of 42 years (range=9 days to 75 years). Distribution of isolation of *Enterobacter* spp. from different clinical samples was as follows: respiratory tract infection (RTI) (n=49, 51%), skin and soft tissue infection (SSTI) (n=18, 18.8%), urinary tract infection (UTI) (n=12, 12.5%), bloodstream infection (BSI) (n=7, 7.3%), abdominal infection (n=5, 5.2%), and eye infection (n=5, 5.2%). Moreover, the recovered *Enterobacter* isolates from Intensive Care Unit (ICU), Internal, Surgery, and Transplantation wards were 55 (57.3%), 36 (37.5%), 4 (4.2%), and 1 (1%), respectively. All 96 clinical isolates of *Enterobacter* were classified as *E. gergoviae* (n=52, 54.2%),* E. aerogenes *(n=34, 35.4%), *E. cloacae* (n=7, 7.3%), and *Cronobacter (E) sakazakii* (n=3, 3.1%). 

***Antimicrobial resistance among Enterobacter isolates***

The results of susceptibility testing are depicted in Table 1. All 96 clinical isolates revealed resistance to all antimicrobials with different proportions. The highest resistance (non-susceptible isolates) rate was seen to β-lactams, including ampicillin, amoxicillin-clavulanate, cefoxitin, and ceftazidime. Conversely, the lowest resistance rate was against imipenem (29.2%), followed by amikacin (30.2%). Among different *Enterobacter* spp., *E. gergoviae* represented the highest (90%) resistance to antimicrobial agents. The majority of isolates (n=93, 96.9%) exhibited a multi-drug resistant (MDR) phenotype. Except for *E. gergoviae *isolates whose MDR rate was 94.2%, all the remaining isolates from other species were MDR. 

Totally, among 96 *Enterobacter* isolates, 35 (36.4%) were positive for the ESBL phenotype. The prevalence of ESBL in* E. gergoviae*,* E. aerogenes*, *E. cloacae*, and *C. sakazakii* was 28.8% (15/52), 50% (17/34), 28.6% (2/7), and 33.3% (1/3), respectively. Among the antimicrobial agents evaluated, imipenem was the most active antibiotic (80%) against the ESBL-positive isolates, and ciprofloxacin had a notable *in vitro* activity (68.6%). There was no significant correlation between ESBL production and higher antibiotic resistance, except for ceftazidime (Table 1). All ESBL producers were MDR; however, compared to non-ESBL producers (95.1%) the differences were not statistically significant (*P*=0.18). 

***Characterization of ESBL and carbapenemase genes***

Of the 35 isolates identified as ESBL-producers, 16 (45.7%) isolates harbored the TEM type enzyme, and 15 (42.8%) and 8 (22.8%) carried CTX-M and SHV type enzymes, respectively. A statistically significant difference was determined between ESBL-positive isolates and the presence of TEM, CTX-M, and SHV genes with values 0.021, 0.004, and 0.02, respectively. *bla*_TEM_+ *bla*_CTX-M_ was found to be the frequent combination (n=9, 9.4%), followed by *bla*_TEM_+*bla*_SHV_+*bla*_CTX-M_ (n=3, 3.1%) (Table 3). Among ESBL-producers, *bla*_IMP_ and *bla*_NDM_ genes were sought in 2 (5.7%) and 5 (14.3%) of the isolates, respectively. Furthermore, there was no significant correlation between any of the mentioned genes among ESBL-producing isolates. 

Of the 96 *Enterobacter* spp., 28 (29.2%) were phenotypically non-susceptible carbapenem isolates (Table 1); however, 3 (3.1%) and 8 (8.3%) of them harbored *bla*_IMP_ and *bla*_NDM_ genes. No PCR products were detected for any of the *bla*_VIM_ and *bla*_KPC_ genes investigated (Table 2). Meanwhile, sequencing results confirmed that all of the tested *bla*_NDM_ positive isolates were NDM-1 variant.

## Discussion

An increase in the emergence of MDR *Enterobacter* spp. producing ESBLs and carbapenemases has limited therapeutic options. Therefore, to reduce the mortality of nosocomial infections caused by these species, their early identification is necessary (17, 18). In the present study, we characterized the antimicrobial resistance pattern and the presence of seven ESBL and carbapenemase genes among 96 clinical isolates of *Enterobacter* recovered from an Iranian population. In the literature, *E. cloacae* and *E. aerogenes* have been suggested as the most common species of *Enterobacter* (1, 8). In our survey, by contrast,* E. gergoviae *was found the most frequently isolated species (54.2%), followed by *E. aerogenes, E. cloacae*, and *C. sakazakii* with frequencies 35.4%, 7.3%, and 3.1%, respectively. To our knowledge, there has been no further report of this species as an emerging nosocomial pathogen until this work in Iran. But in studies in Germany, Spain, and Hong Kong, *E. gergoviae* was isolated from clinical samples with frequencies of 26.1%, 6.6%, and 2.9%, respectively (19-21). In another survey from a nosocomial outbreak of bacteremia, 11 *E. gergoviae *were isolated from 11 babies in neonatal ICU (NICU) (22).

*Enterobacter* spp. are responsible for a wide variety of nosocomial infections, particularly wound infections, bacteremia, and pneumonia (1, 23). In the current study, most isolates (51%) were recovered from RTIs. Consistent with our work, Qin and co-workers (11) and Hoffmann *et al.* (17) isolated 91% and 37.8% of strains from respiratory tract samples, respectively. In contrast, in several studies from Brazil (7), China (24), a global surveillance program (25), and Korea (8), blood and abdominal samples were the most common sites of *Enterobacter* isolation. In our study, 57.3% of isolates were obtained from the ICU ward. Likewise, two authors from Germany (17) and Spain (26) showed most strains were isolated from ICU.

Members of ESBL-producing and CRE, including *Enterobacter* spp., have been emerging and increasing around the world and become a matter of great concern (11, 23, 27). By analysis of susceptibility testing, it is found the majority of our isolates were remarkably resistant to most of the antimicrobials tested, with 96.9% of strains showing MDR phenotype, making them a public health concern in our area. This finding does not coincide with two previously reported works from Iran with prevalence of 17.5% and 47.5% (28, 29). Carbapenem resistance was defined as resistance to one or more carbapenems according to CLSI guidelines (7). In the current study, 29.2% of isolates were non-susceptible to imipenem (carbapenem-resistant). In several studies from different areas, these rates were reported 8.7%, 25.7%, 35.1%, 18.3%, and 5.1% (8, 23, 28, 30, 31). Although CRE isolates are usually extensively drug-resistant, some isolates may be still susceptible to amikacin and ciprofloxacin. Hu and co-workers reported the rate of susceptibility of their isolates to amikacin and ciprofloxacin were 10.4 and 13%, respectively (32). Instead, 69.8% and 63.5% of our isolates were fortunately susceptible to amikacin and ciprofloxacin, correspondingly, indicating an alternative choice to treatment of infections caused by *Enterobacter* resistant isolates, especially ESBL-producers. 

Thirty-five (36.4%) of our isolates were ESBL-producers using the phenotypic tests. The result was less than those observed by two other studies from Iran with prevalence of 52.6% and 44.2%, respectively (28, 29). The use of antimicrobials, including cefoxitin and ceftazidime in Iran, could partly explain this slightly high rate of ESBL among* Enterobacter* isolates. In agreement with our findings, in two investigations performed in Korea (33) and Germany (17), 35.4% and 40% of *E. cloacae* were ESBL-positive, respectively; however, Villa and colleagues (31) and Yu *et al.* (34) detected only 5.1% and 15% of isolates as ESBL-producers, correspondingly. On the other hand, in a report from China, ESBL-producing *Enterobacter* isolates comprised 65.7% (23). These discrepancies might be due to the differences in the epidemiology of isolates or sample sizes of studies.

It has been suggested that CTX-M and SHV-type beta-lactamases have been the predominant ESBLs in *Enterobacter* spp. (35). Conversely, beta-lactamases belonging to the TEM (31.3%) family were the ESBLs encountered most frequently in our isolates, followed by CTX-M (26%) and SHV (12.5%) types. Likewise, Ghanavati and colleagues reported *bla*_TEM_ and *bla*_SHV_ as the most and less prevalent ESBL genes in their *Enterobacter* species (28). In a study from Brazil, *bla*_TEM-1 _and *bla*_CTX-M _were the frequently identified ESBL genes with no *bla*_SHV _among *E. aerogenes* and *E. cloacae* isolates studied (7). In several studies from Algeria, Spain, and Korea, high rates of the CTX-M type with frequencies of 76%, 52.3%, 53.3%, and 60.8% have been reported, respectively (12, 26, 33, 36). Conversely, in an investigation from England (37), in none of *Enterobacter* spp. isolated from blood and urine samples, the *bla*_CTX-M _gene was detected. This result is not in agreement with our findings. The rate *bla*_SHV _was observed in our study was similar to another study with frequency of 10% (12), but much lower than those identified (52%) in Korea (36). On the contrary, in a work from Spain (26), no *bla*_SHV_ and *bla*_TEM _were identified among *Enterobacter* obtained isolates. 

Among evaluated carbapenemase genes, only *bla*_NDM _(n=8, 8.3%) and *bla*_IMP_ (n=3, 3.1%) were detected. In other words,* bla*_NDM _was the most prevalent MBL as a mechanism of resistance to carbapenems in our *Enterobacter* spp. This study is the second reported presence of *bla*_NDM _among clinical isolates of *Enterobacter* in Iran. While in the first report 2.5% of isolates were carried *bla*_NDM-1_, the species and origin of isolates were not mentioned (30). In our work, 6 (75%) NDM-positive isolates were related to *E. gergoviae, *which is the first report of this species in Iran, and two other cases belonged to *E. aerogenes *and *E. cloacae*. This result is not consistent with those published in other countries such as China (23), Spain (38), Korea (8), and Mexico (9) with frequencies of 2.8%, 0%, 0%, and 100%, respectively, where *bla*_NDM _had been identified in *E. aerogenes *and/or *E. cloacae*.

*bla*_KPC_ has been reported as the predominant carbapenemase gene associated with CRE intrahospital infections (9). The importance of KPC enzymes is due to high-level resistance to all beta-lactams and distinct levels of resistance to the carbapenem antibiotics (39). A study in Brazil showed 88.6% of *E. aerogenes* and 100% of *E. cloacae* isolates harbored *bla*_KPC_, and 8 other carbapenemase genes evaluated were not detected in any isolate (7). In an investigation in the United States, 11 (25%) isolates of the 44 ertapenem-nonsusceptible *Enterobacter* isolates were found to be KPC-producer (40). In our study, by contrast, no *Enterobacter* isolates harboring *bla*_KPC_ were diagnosed. This result is consistent with results from other researchers who reported the rate of 0% for the *bla*_KPC_ gene (8). However, in the studies from China and Spain, the frequencies of 19.3% and 6.8% were determined, respectively (11, 31).

It has been mentioned that carbapenemase production is mostly related to the presence of VIM and IMP types (23). Indeed, VIM-1-producing *Enterobacter* isolates, especially *E. cloacae*, have been frequently reported in some European countries and particularly in Spain and become major nosocomial pathogens in southern Europe and Asia (31, 38). In our investigation, however, no isolate carrying *bla*_VIM_ was found and only 3.1% of isolates (3 *E. aerogenes* isolates) harbored the *bla*_IMP_ gene. In accordance with the literature, 52% and 100% (7 isolates) of *E. cloacae* isolates in two studies from Spain were found to be *bla*_VIM_ producers (31, 38). On the other hand, in research from the Far East the rates of *bla*_IMP_ (0.5%) and *bla*_VIM_ (0.25%) were reported rare (8), similar to our findings. Furthermore, in a recent work from Iran, no carbapenemase gene was detected among clinical isolates of *Enterobacter* spp. (28). Taken together, these discrepancies in results are probably due to the distribution of geographically different regions and genetic heterogeneity of strains. 

A limitation of the current study is the relatively small sample size. Another limitation of the work is that we could not evaluate the presence of other ESBL and carbapenemase genes from different classes of beta-lactamases to better assess beta-lactam resistance in our isolates. 

**Table 1:** Distribution of antibiotic resistant *Enterobacter* isolates according to ESBL production

**Antibiotic**	**Total (N = 96)** **No. (%)**			**ESBL-Positive (N = 35)** **No. (%)**			***P*** **-value ** ^a^
	**R**	**I**	**S**	**R**	**I**	**S**	
**Ampicillin**	90 (93.8)	5 (5.2)	1 (1)	31 (88.6)	4 (11.4)	0	0.45
**Amoxicillin-clavulanate**	84 (87.5)	6 (6.3)	6 (6.3)	30 (85.7)	2 (5.7)	3 (8.6)	0.48
**Cefoxitin**	80 (80.3)	7 (7.3)	9 (9.4)	30 (85.7)	1 (2.9)	4 (11.4)	0.6
**Ceftazidime**	70 (72.9)	2 (2.1)	24 (25)	30 (85.7)	2 (5.7)	3 (8.6)	0.005
**Imipenem**	21 (21.9)	7 (7.3)	68 (70.8)	5 (14.3)	2 (5.7)	28 (80)	0.13
**Gentamicin**	39 (40.6)	1 (1)	56 (58.3)	14 (40)	0	21 (60)	0.8
**Amikacin**	22 (22.9)	7 (7.3)	67 (69.8)	6 (17.1)	6 (17.1)	23 (65.7)	0.51
**Trimethoprim** **sulfamethoxazole**	45 (46.9)	6 (6.3)	45 (46.9)	19 (54.3)	4 (11.4)	12 (34.3)	0.061
**Nitrofurantoin**	68 (70.8)	14 (14.6)	14 (14.6)	22 (62.9)	5 (14.3)	8 (22.9)	0.082
**Ciprofloxacin**	31 (32.3)	4 (4.2)	61 (63.5)	8 (22.9)	3 (8.6)	24 (68.6)	0.44

^a^ Compared with susceptibility rates of ESBL-negative isolates

R: resistant; I: intermediate-resistant; S: susceptible

**Table 2:** Distribution of ESBL and carbapenemase genes among *Enterobacter* spp

**Species**	**ESBL genes** **No. (%)**			**Carbapenemases genes** **No. (%)**			
	CTX-M	TEM	SHV	IMP	NDM	VIM	KPC
*E. gergoviae* (N = 52)	14 (26 .9)	19 (36.5)	9 (17.3)	0	6 (11.5)	0	0
*E. aerogenes* (N = 34)	8 (23.5)	8 (23.5)	2 (5.9)	3 (8.8)	1 (2.9)	0	0
*E. cloacae* (N = 7)	2 (28.6)	2 (28.6)	1 (14.3)	0	1 (14.3)	0	0
*C. sakazakii *(N= 3)	1 (33.3)	1 (33.3)	0	0	0	0	0
Total (N = 96)	25 (26)	30 (31.3)	12 (12.5)	3 (3.1)	8 (8.3)	0	0

**Table 3:** Resistance genes pattern identified among *Enterobacter *isolates

**Gene pattern**	**Frequency**	**Percent**
No gene	50	52.1
TEM	10	10.4
SHV	4	4.2
CTX-M	7	7.3
NDM	1	1.0
TEM/SHV	2	2.1
TEM/CTX-M	9	9.4
TEM/IMP	2	2.1
TEM/NDM	1	1.0
SHV/NDM	1	1.0
CTX/NDM	2	2.1
TEM/SHV/CTX-M	3	3.1
TEM/CTX-M/IMP	1	1.0
TEM/CTX-M/NDM	1	1.0
SHV/CTX-M/NDM	1	1.0
TEM/SHV/CTX-M/NDM	1	1.0
**Total**	96	100.0

## Conclusion

ESBL-positive and carbapenem-resistant *Enterobacter *spp., particularly *E. gergoviae *have become a concern in our area. With respect to the findings, amikacin may still be suitable for treatment of infections caused by MDR *Enterobacter* isolates. Additionally, educational programs for healthcare workers about diminishing risk of transmission of *Enterobacter* isolates as serious nosocomial pathogens should be implemented in our hospitals. 
